# Radium in the Treatment of Deafness

**Published:** 1914-03-28

**Authors:** H. D. McColloch


*To the Editor of* The Hospital.

### Radium in the Treatment of Deafness.

*To the Editor of* The Hospital.

Sir,—I am indebted to your management for a copy of your article on the therapeutic application of radiations, which purports to be a collection of expert views upon the facts of radio-therapy. The striking feature of this (you observe) "is the wide divergence of opinion as to its effects in different diseases." I have no desire to criticise the generalisations made in that article, but it is with much reluctance that I feel constrained to address you with reference to the statement on p. 680, which is entirely misleading, if not untrue. That statement runs: "A good many sensational reports of cures of deafness by radium and by *x*-rays have been in circulation this last year or two. It seems to be the unanimous opinion of otologists that these reports have no substantial foundation. Both radium and ultra *x*-rays are authoritatively described as total failures by experts in this specialty." This unwarranted statement is one that I cannot allow to pass unchallenged. To controvert it you will perhaps permit me to cite the President of the Otological Section of the Royal Society of Medicine, Mr. Richard Lake, who in 1910 put (consciously or unconsciously) my previous experimental observations to a crucial test on a case of otosclerosis, which had resisted every form of treatment known to him during two years. If his statements are to be credited, the results in that first and (so far as can be gathered) only experience of his, so surpassed his expectations that he made it the subject of his contribution to the proceedings of the Ninth International Otological Congress held at Boston, U.S.A., in August 1912. To this volume I have much pleasure in referring you. I will not take up your space by making specific citations from it. In so far as the efficacy of radio-therapy is concerned it is amply eloquent.

Again, I am able to state that, after the application of this method of ultra *x*-ray irradiation to four crucial cases of chronic labyrinthine deafness (otosclerosis), all of which were otologically independently tested by the Hon. Secretary of the British Oto-laryngological Society, both previous to and after a three months' course of irradiation (March to June last), they were pronounced to have unmistakably improved by the same authority in every case; especially in the most hopeless one of the four. These patients were exhibited at a clinical meeting of that Society at the rooms of the Medical Society of London on June 16 last. So "sensational" and convincing were these results that I was requested by the Fellows to read a paper on the subject to them at their next meeting. Not only was this done on October 29 last, but the complete text of the paper, along with a list of sixty-four cases of similar hopeless deafness treated by me during the two preceding years, was printed and circulated to the members a week before that meeting. This was done in order to invite adequate discussion and criticism. Owing to the fact that otologists are admittedly quite unfamiliar with radio-therapy, just as dermatologists were, and gynæcologists still are, I requested the invitation of some medical physicists, as well as such an authority as Dr. W. S. Lazarus-Barlow, to the meeting; but this was not done. A report of the discussion in brief was published in the *Lancet* on November 15 last.—I am, yours faithfully, H. D. McCulloch, M.B., C.M.

[The concluding paragraphs of the above letter have been omitted; they are sarcastic in tone and irrelevant to the argument. As for the latter, we would refer our correspondent to the *Journal of Laryngology, Rhinology, and Otology*, February 1914, p. 112, where he will find the authoritative pronouncements of experts upon which the paragraph he objects to was based.—Ed. The Hospital.]

### The Children and Homœopathy.

*To the Editor of* The Hospital.

